# Nordic diet and its benefits in neurological function: a systematic review of observational and intervention studies

**DOI:** 10.3389/fnut.2023.1215358

**Published:** 2023-08-14

**Authors:** Reyhaneh Sadat Jafari, Vahideh Behrouz

**Affiliations:** ^1^Student Research Committee, Kerman University of Medical Sciences, Kerman, Iran; ^2^Department of Nutrition, Faculty of Public Health, Kerman University of Medical Sciences, Kerman, Iran

**Keywords:** diet, Nordic, brain, neuroprotection, cognition

## Abstract

**Introduction:**

Neurological disorders have been considered the major contributors to global long-term disability and lower quality of life. Lifestyle factors, such as dietary patterns, are increasingly recognized as important determinants of neurological function. Some dietary behaviors, such as Nordic diet (ND) were likely to have protective effects on brain function. However, an understanding of the effectiveness of the ND pattern to improve neurological function and brain health is not fully understood. We review the current evidence that supports the ND pattern in various aspects of neurological function and addresses both proven and less established mechanisms of action based on its food ingredients and biochemical compounds.

**Methods:**

In this systematic review, PubMed, Web of Science, and Scopus databases were searched from inception to February 2023. Observational and intervention studies were included.

**Results:**

Of the 627 screened studies, 5 observational studies (including three cohorts and two cross-sectional studies) and 3 intervention studies investigating the association between ND and neurological function. Observational studies investigated the association of ND with the following neurological functions: cognition, stroke, and neuropsychological function. Intervention studies investigated the effects of ND on cognition and depression.

**Discussion:**

Despite the limited literature on ND and its association with neurological function, several aspects of ND may lead to some health benefits suggesting neuroprotective effects. The current state of knowledge attributes the possible effects of characteristic components of the ND to its antioxidant, anti-inflammatory, lipid-lowering, gut-brain-axis modulating, and ligand activities in cell signaling pathways. Based on existing evidence, the ND may be considered a recommended dietary approach for the improvement of neurological function and brain health.

**Systematic review registration:**

[https://www.crd.york.ac.uk/prospero/], identifier [CRD2023451117].

## 1. Introduction

Neurological disorders have been considered the major contributors to global long-term disability and lower quality of life (1). Recent reports have documented an alarming increase in the global population living with brain health disorders such as depression, anxiety, migraine, multiple sclerosis, Parkinson’s disease (PD), and Alzheimer’s disease (AD) (2–5). A neurological disorder is any illness of the body’s nervous system including biochemical, structural, or electrical abnormalities in the peripheral nerves, spinal cord, or brain which leads to a wide range of complications. Examples of complications include weakness, confusion, loss of sensation, chronic pain, seizures, and poor coordination (6). Considerable evidence has reported that oxidative and nitrosative stress, neuroinflammation, and mitochondrial dysfunction play a crucial role in the occurrence and progression of these disorders (7). Moreover, cardiometabolic conditions such as obesity, insulin resistance, metabolic syndrome, type 2 diabetes, cardiovascular diseases, and hypertension are involved in some brain health problems and cognitive performance (8).

Diet is increasingly recognized as an important determinant of neurological function. Animal studies demonstrated that nutrition can be involved in neurological function. Some dietary behaviors such as a Western diet, which contains high amounts of saturated fat, refined sugars, and processed foods, may have an interaction with impairment of learning and memory (9, 10). On the other hand, diverse aspects of diet and dietary patterns including the Mediterranean diet (MD), vegetarian diet, and Nordic diet (ND) could be important protective factors for cognitive health (11–14).

The ND, also understood as the Baltic Sea diet, originated in Nordic or Northern European countries including Denmark, Norway, Sweden, Iceland, and Finland (15). A high ND score indicates a high intake of different healthy food items including vegetables, legumes, whole grains (rye, oat, and barley), fruits (berries, apples, and pears), low-fat dairy, rapeseed oil, low-fat types of meat (game and poultry), shellfish, fatty fish (mackerel, herring, and salmon), seafood, salt restriction, and also low intake of sugar-sweetened products as well as moderate consumption of alcohol (16, 17). In the Danish population, previous research has demonstrated that a higher ND score is related to an 11% lower mortality rate (18). Further observational studies also indicated an inverse relationship between adherence to the ND and risk of abdominal obesity (19), markers of inflammation (20), and cardiometabolic risk indicators (17, 21, 22).

Considering that better cardiometabolic status is related to a lower risk of vascular dementia, the ND can decrease the rate of cognitive decline with aging (23). Moreover, the majority of ND features have previously been related to preserved cognition. For example, ND is characterized by the abundant use of berries, providing different types of antioxidants, flavonoids, and other bioactive compounds (24). A 2-year multi-domain lifestyle intervention has shown high berry consumption had a positive effect on cognitive performance compared with no berry consumption (25). Other main components of the ND are rye, oat, and barley; providing high fiber contents. A rapeseed oil-based diet high in α-linolenic acid has been postulated to protect against cognitive decline (24).

A comprehensive search of scientific literature in electronic databases did not find any ongoing or published systematic reviews on a particular type of diet, namely the Nordic diet, and its effects on neurological function. As a result, the primary goal of this research is to conduct a review of scientific literature to determine how ND affects neurological function. Also, the purpose of this review is to provide a summary of the primary molecular mechanisms that are proposed to explain the relationship between the ND, its components, neurological function, and brain health.

## 2. Methods

A comprehensive search was conducted up to February 2023 using Medline/PubMed, Scopus, and Web of Science to recognize observational and intervention studies reporting the effects of ND on neurological function. The search focused on the following terms: (Nordiet OR “Nordic dietary pattern” OR “Nordic diet” OR “Baltic Sea dietary pattern” OR “Baltic Sea diet”) AND (mental OR cognitive OR cognition OR memory OR “neurocognitive disorders” OR aging OR depression OR anxiety OR Alzheimer’s disease OR stroke OR dementia OR brain OR Parkinson’s disease OR migraine OR “multiple sclerosis” OR “neurological diseases”). The search was confined to English language publications, covering all years available in the database. There was no age limit. Moreover, reference lists of retrieved publications were manually searched for relevant articles. The abstract was reviewed to select relevant articles.

Studies that reported the effect of (intervention studies) or the association (observational studies) of the Nordic diet (as exposure) on neurological function (as outcome) were considered for review.

The inclusion criteria were as follows: (a) assessment of the ND as main exposure; (b) studies focusing on neurological function and disorders, cognitive spectrum (including cognition, memory, attention span, learning), and psychosocial and emotional aspects (including depression and anxiety) were included; (c) enrolled humans at all ages; (d) analytical epidemiological studies, i.e., observational studies (prospective cohort studies, case–control, or cross-sectional) and intervention studies (randomized control trials, and non-randomized trials, i.e., pre-post studies); and (e) studies written in English and published in reputable journals.

The exclusion criteria were: (a) no original studies (e.g., editorials, review articles, non-research letters); (b) case series or case reports; (c) lack of data on the ND (e.g., investigated only specific nutrients-rich in the ND); and (d) studies not conducted in humans. Moreover, intervention studies that utilized lifestyle interventions alongside the ND intervention (such as behavioral management or exercise) were excluded.

EndNote referencing software (version X8, Thomson Reuters) was used to import identified publications, and two authors (RJ and VB) independently examined the titles and abstracts of those publications. Full-text articles were retrieved if a suitable record was found. Any disagreements and discrepancies were addressed and resolved by consensus between the authors. The reference lists of selected records and review articles were checked for additional potentially relevant articles following the retrieval of full-text studies.

**Figure 1:**
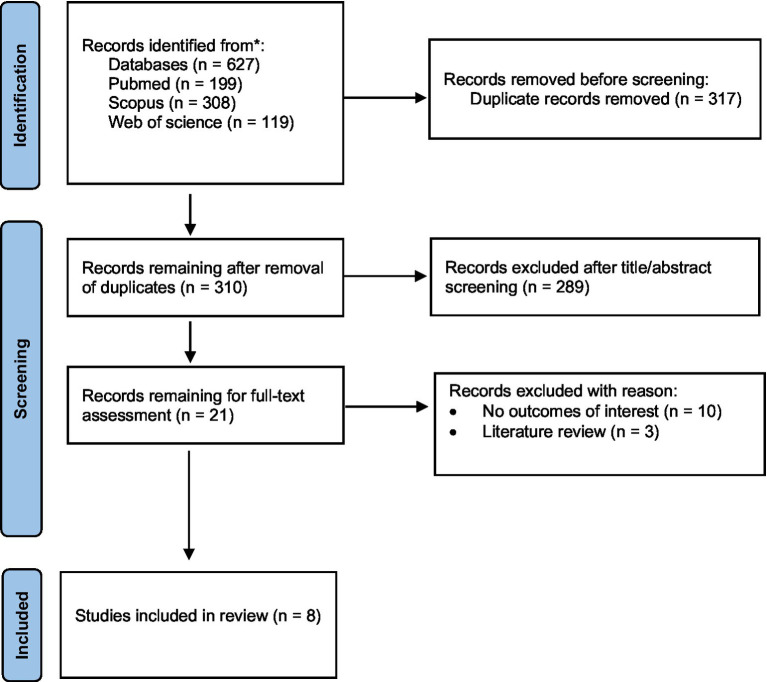
The study selection flow diagram.

Data extraction was done by two authors (RJ and VB) on the included articles using a predefined data extraction form. The following data were extracted: first author, publication year, country, study design, duration, participants and sample size, dietary assessment method, details of control and intervention groups (intervention studies), outcome assessment, and main findings. Discrepancies in data extraction were addressed and resolved by consensus between the authors (Figure 1).

## 3. Nordic diet

The ND is designed to provide better health-related quality of life. As its name implies, the ND is a dietary pattern focusing on the consumption of traditional, organic, plant-based, and local foods originating from the Scandinavian countries including Iceland, Finland, Norway, Sweden, and Denmark (26). It is also known as the Baltic Sea diet which is recommended for healthy subjects to introduce healthier options for eating according to foods that are commonly available in the Scandinavian countries (27, 28).

**Figure 2:**
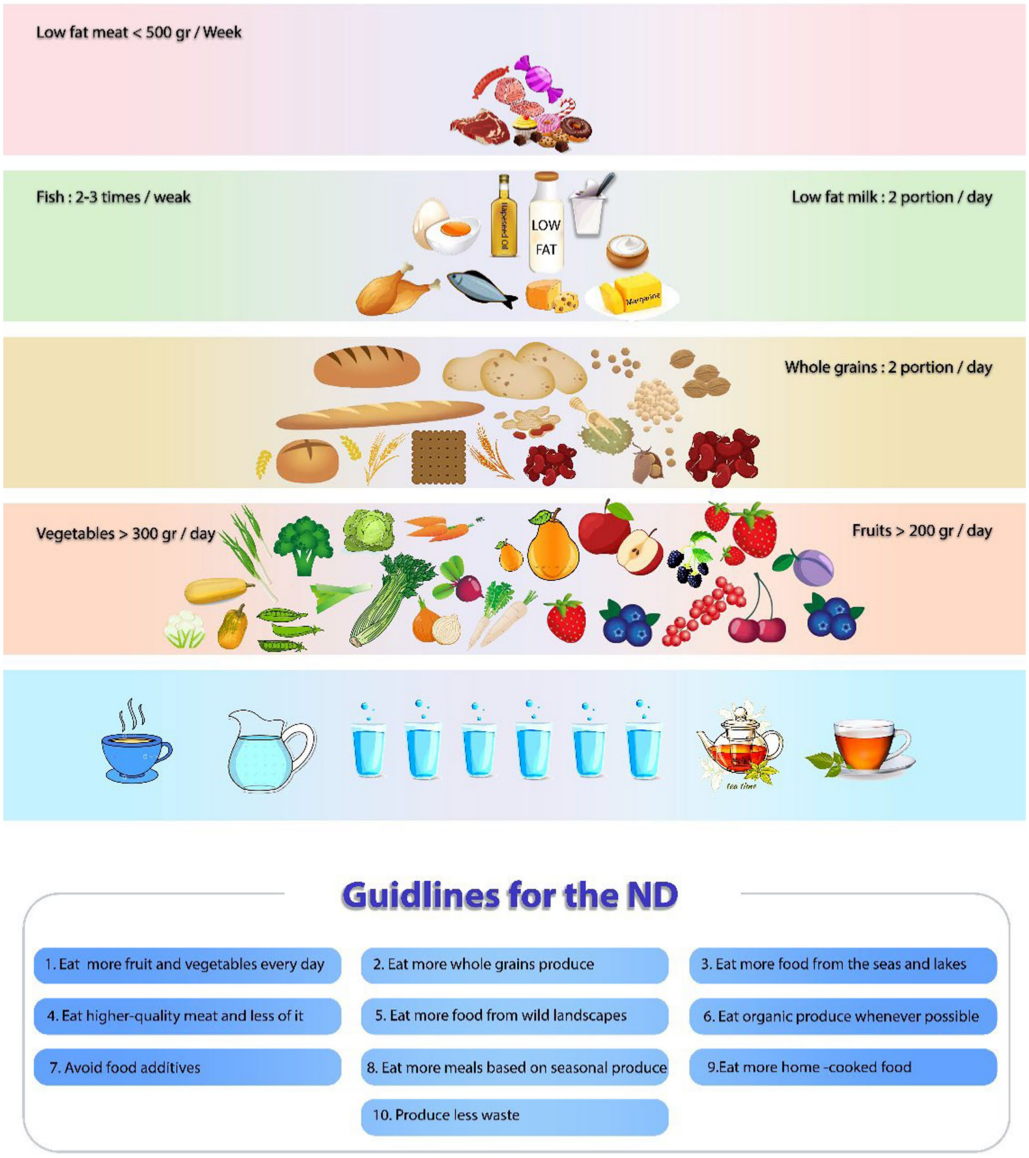
The pyramid illustrates the overall characteristics of the Nordic diet. The main components of the ND including Nordic vegetables, roots, cabbages, peas, Nordic fruits, apples, pears, and berries, are placed at the bottom of the pyramid. Whole-grain cereals (rye, oats, and barley), potatoes, legumes, nuts, and seeds are placed in the middle of the pyramid. Above them are located fatty fish, low-fat or fat-free dairy products, low-fat choices of meat (poultry and game), egg, margarine, and rapeseed oil. Foods that should be consumed carefully, such as animal fats (butter), red and processed meat (beef, pork, processed meat products, and sausage), sweets, and chocolate, are at the top of the pyramid.

The new ND, a gastronomic interpretation of the ND, was designed in 2004 by a group of nutritionists, scientists, and leading Nordic chefs to increase the focus on local cuisine and promote the excellence of Nordic food internationally (26, 29). It is based on four crucial principles: sustainability, health, Nordic identity, and gastronomic potential (30). The new ND Manifesto emphasizes wild, foraged, local, fresh, and very tasty foods. Generally, this dietary pattern includes less fat, less sugar, less alcohol, high fiber, and high in fish and seafood. There are some foods and ways to prepare them that are the same in Scandinavian countries, including berries, apples, cabbage, peas, root vegetables, barley, oats, rye, fermented milk, fat-free or low-fat dairy products, fish (salmon, Baltic herring, and mackerel), and rapeseed oil consumption. It also suggests an increase in legume consumption, which increases total protein intake, but processed meat products and red meat are consumed less (31, 32). Moreover, a critical part of the diet in the Scandinavian nations is fish and other seafood, due to their rich marine archipelago (30, 33). Considering the geographical location of the Nordic nations and the growing conditions of rapeseed plant in the winter, rapeseed oil (also recognized as canola oil) is the main source of fats in the ND (34). Water is generally recommended as a drink in the ND, and salt intake is limited to 5–6 gr/day. Moreover, in some Scandinavian nations, taking a vitamin D supplement is advised as a dietary supplement (35–37). More information on the components of the ND is given in Figure 2.

After decades of collaboration among the Scandinavian countries, guidelines for dietary composition and reference values for nutrient intake were published through the joint publication of the Nordic Nutrition Recommendations (NNRs). Based on NNRs, the amount of macronutrient distribution range (as calorie percent) is 10–20% of total energy intake should be derived from proteins, 45–60% from carbohydrates, and 25–40% from fats (34, 38). In terms of dietary recommendations, the ND has similarities with the MD, both recommend healthy foods for the general population, that are rich in vegetables, fruits, legumes, nuts, seeds, whole-grain cereals, and seafood, and low in red meat and processed foods (39). The notable point difference between these dietary patterns is the type of oil used in each dietary pattern. The ND is based on rapeseed oil (more polyunsaturated fat), while the MD mainly recommends olive oil (34).

The ND is also similar to the Finnish Geriatric Intervention Study to Prevent Cognitive Impairment and Disability (FINGER) study which investigated whether diet, vascular management, exercise, and cognitive training could improve cognitive function in at-risk older adults (40). It emphasizes fruits, vegetables, whole grains, and fish while limiting red meat, sucrose, and saturated fats. The FINGER trial showed that this comprehensive program could increase white-matter integrity and improve cognition, executive function, and processing speed in older adults with elevated dementia risk (41, 42).

A growing body of evidence suggests that the components of ND are related to beneficial health effects, but little is recognized about the overall health-related impacts of ND (43, 44). Several studies reported that the ND is related to improving cardiovascular risk indicators including hypertension and dyslipidemia (45, 46). Several observational and intervention studies have indicated an inverse association between adherence to the ND and risk of type 2 diabetes mellitus, colorectal cancer, stroke, inflammation, coronary heart disease, and all-cause mortality (22, 47–51). So, the health benefits of the ND have increasingly become an area of interest.

## 4. Nordic diet and neurological function: current evidence

To date, a few studies have examined the role of the ND in neurological diseases. There are five observational studies (including three cohorts and two cross-sectional studies) and three intervention studies investigating the association between the ND and neurological function (Table 1).

**Table 1:** Characteristics of observational and intervention studies.

First author	Country	Study design	Duration	Participants (*n*)	Dietary assessment method	Intervention and control groups	Main findings
Mannikko et al. 2015 (24)	Finland	Cohort	4 years	Men and women aged 57–78 years (*n* = 1,140)	4-day food records	–	High adherence to ND: ↑ Cognitive function
Hansen et al. 2017 (22)	Denmark	Cohort	13.5 years	Men and women aged 50 to 64 years (*n* = 55,338)	FFQ	–	High adherence to ND: ↓ Risk of stroke
Haapala et al. 2015 (52)	Finland	Cross-sectional	–	Children aged 6–8 years (*n* = 428)	4-day food records	–	Low adherence to ND: ↓ Cognition in boys
Sørensen et al. 2015 (53)	Denmark	RCT (cross-over)	3 months	Children aged 8–11 years (*n* = 726)	7-day web-based dietary assessment software	Intervention group: NDD school meals Control group: usual school meals	↑ School performance ↑ Reading comprehension ↑ LCPUFA status ↔ Ferritin and Hb
Sørensen et al. 2015 (54)	Denmark	RCT (cross-over)	3 months	Children aged 8–11 years (*n* = 693)	7-day web-based dietary assessment software	Intervention group: NDD school meals Control group: usual school meals	↔ Concentration performance ↑ Reading speed
Sabet et al. 2021 (55)	Sweden	RCT	8 days	Depressed and non-depressed subjects (*n* = 16)	Meal evaluation questionnaire	Intervention group: ND Control group: control diet	↓ Depressive symptoms
Abbaszadeh et al., 2020 (56)	Iran	Cross-sectional	–	Female students aged 18–25 (*n* = 181)	FFQ	–	High adherence to ND: ↓ Stress and anxiety score ↑ Quality of life score
Shakersain et al., 2018 (57)	Sweden	Cohort	6 years	Dementia-free adults aged ≥60 (*n* = 2,223)	FFQ	–	Moderate to high adherence to ND: ↓ Cognitive decline

FFQ, Food frequency questionnaire; ND, Nordic diet; NDD, New Nordic diet; LCPUFA, Long-chain polyunsaturated fatty acids; Hb, Hemoglobin.

### 4.1. Observational studies

#### 4.1.1. Cognition

In 2015, a 4-year study involving 1,140 women and men aged 57–78 years with normal cognition was done to estimate the cross-sectional and longitudinal associations of the ND with cognitive performance at baseline and 4-year follow-up in a Finnish population-based random sample. Assessment of cognition by the Consortium to Establish a Registry for Alzheimer’s Disease (CERAD) neuropsychological battery and the Mini-mental State Examination (MMSE) indicated that subjects with better adherence to the ND experienced higher scores in global cognitive functioning over a four-year study period compared to baseline after adjustment for lifestyle and demographic confounders (24). A population cohort study on 2,223 dementia-free adults aged ≥60 followed up for 6 years reported that moderate and high adherence to the ND was more firmly connected with a better-preserved cognitive performance, assessed by MMSE, among Swedish older adults compared to low adherence to the ND (57). Another cross-sectional study including 428 children aged 6–8 years demonstrated that lower scores of the Baltic Sea diet, based on 4-day food records, were related to lower cognitive performance (assessed by Raven’s Colored Progressive Matrixes) among children in Finland (52).

#### 4.1.2. Stroke

A Danish cohort study among 55,338 men and women aged 50 to 64 years with a median follow-up of 13.5 years revealed that adhering to a healthy ND, evaluated with a healthy Nordic food index score, was related to a lower risk of ischemic stroke in the Danish Diet, Cancer, and Health cohort (22).

#### 4.1.3. Neuropsychological function

A cross-sectional study containing 181 female students aged between 18 and 25 years was conducted to examine the association between adherence to the health-promoting ND with neuropsychological activity. A collection of standardized questionnaires such as insomnia severity index, quality of life, depression anxiety stress scale, cognitive ability questionnaire, and Epworth sleep scale was used to evaluate the neurological function determinants such as anxiety, stress, memory, and sleep disorders. Analyses of data reported that adherence to the ND was inversely associated with anxiety and stress scores and directly linked to health-related quality of life (56).

### 4.2. Intervention studies

#### 4.2.1. Depression

A 8-day randomized controlled pilot trial conducted in Sweden involving 16 women and men aged between 18 and 65 years with major depressive disorder in a controlled setting with provided foods, demonstrated that a healthy ND compared to a control diet was related to a greater reduction in depressive symptoms (55). Studies with a stronger methodology, that consider a longer period and larger sample size, may help to confirm the findings of this study.

#### 4.2.2. Cognitive

A school meal, randomized, controlled, cross-over trial involving 693 Danish children aged 8–11 years was conducted to investigate the effects of optimal well-being, development, and health for Danish children (OPUS) through a healthy new Nordic school meal on the concentration and school performance for 3 months. A healthy Nordic school meal program did not affect concentration function compared to the usual packed lunch from home but increased reading speed (54). Secondary analyses of the OPUS school meal study on 726 Danish children aged 8 to 11 years old indicated that increased fish intake through a healthy Nordic school meal program during 3 months was associated with greater improvements in school performance, reading comprehension, as well as whole blood long-chain polyunsaturated fatty acids status (53).

## 5. Mechanisms linking the ND to neurological function

### 5.1. Vegetables and fruits

The ND is rich in vegetables and fruits which are the main sources of nutritious components such as minerals, natural antioxidants, vitamins, and dietary fibers (58, 59). These components have multiple beneficial effects in preventing and improving several chronic disorders. Their benefits for health are mediated by polyphenolic compounds to provide antioxidant, anticarcinogenic, antimicrobial, antiviral, and anti-inflammatory effects (58, 60–62). Thus, daily consumption of foods rich in plant-derived polyphenols has been encouraged, especially in the Western world (61, 63).

Berries are important and abundant wild fruits in the Nordic countries with significant health-promoting outcomes, attributed mainly to a special class of polyphenols known as anthocyanins (64). Anthocyanins are organic water-soluble glycosidic pigments widely found in dark-colored vegetables, fruits, and grains with considerable inherent antioxidant properties (65). It has been reported that anthocyanins have a wide and various range of beneficial impacts such as anti-inflammatory, immunomodulatory, cardioprotective, hepatoprotective, neuroprotective, anticancer, antidiabetic, anti-obesity, and antioxidant effects, which make them promising factors for the prevention and treatment of pathological circumstances including neurological disorders (66, 67). Multiple lines of evidence suggest that anthocyanins may have neuroprotective and anti-neuroinflammatory activities that result in improving neural function (68). Neuroinflammation defines specific processes related to the trafficking of immune cells in the central nervous system, which leads to a pathological condition by affecting certain pathways. During the neuroinflammatory process, activation of microglia and astrocytes can cause proinflammatory cytokines to be released and, in turn, activate a second level of inflammatory signaling pathways like nuclear factor-kappa B (NF-κB) (69, 70). Thus, oxidative stress and neuroinflammation can disrupt normal brain function (71). It is suggested that increased generation of reactive oxygen species (ROS) may trigger oxidative injury, cellular damage, mitochondrial dysfunction, and impaired deoxyribonucleic acid repair system which are detrimental factors for neurological function (72, 73). Polyphenols, including anthocyanins, can prevent neuroinflammation, protect neurons against neurotoxin-induced damage, and promote cognitive performance, learning, memory, and neuron survival through their anti-inflammatory and antioxidant activities (74–76). In this regard, Poulose et al. demonstrated that anthocyanins protect the microglial cells subjected to lipopolysaccharide (LPS), a bacterial endotoxin, against neuroinflammation by preventing the activation of p38 and NF-κB and reducing proinflammatory factors such as cyclooxygenase-2 (COX-2) and tumor necrosis factor-α (TNF-α) (77). These findings are consistent with another study in which anthocyanins prevented the up-regulation of inflammatory pathways including Phosphoinositide 3-kinase (PI3K)/AKT, NF-κB, and mitogen-activated protein kinases (MAPKs) in microglial cells, decreasing the generation of pro-inflammatory factors, such as TNF-α, nitric oxide (NO), interleukin (IL)-1β, and prostaglandin E2 (PGE2) (78).

It is noteworthy that the relatively low bioavailability of anthocyanins in the brain may limit their efficacy. Indeed, low concentrations of circulating anthocyanins are considered to be due to the metabolism by gut microbiota to produce different phenolic acid and aldehyde metabolites (79). Some studies indicated that protocatechuic acid (PA), a key anthocyanin metabolite, is a neuroprotective factor through anti-inflammatory and antioxidant features (79, 80).

Cruciferous vegetables including kale, cabbage, Brussels sprouts, horseradish, cauliflower, radish, turnips, and broccoli sprouts are also identified as one of the main components in the ND (81). These vegetables contain a wide variety of nutrients such as protein, carbohydrate, vitamins, minerals, phytochemicals (Tannins, flavonoids, anthocyanins, and carotenoids), phytosterols, and non-nutritive metabolites such as nitrogen-containing compounds and sulfur compounds (glucosinolates and S-methyl cysteine sulfoxide) (82). Several studies have examined the health-promoting effects of cruciferous vegetables in humans and showed that higher consumption of these vegetables is related to a reduced risk of several chronic disorders (83). Besides phytochemicals, the beneficial health characteristics of these vegetables are mainly related to the functions of glucosinolates. In case of plant tissue damage, glucosinolates are hydrolyzed by enzymatic activity, and metabolites such as isothiocyanate, nitriles, and thiocyanates are formed. Also, glucosinolates hydrolysis can occur by intestinal microbiota (84, 85). Cruciferous vegetables, due to their glucosinolates and other metabolites, exhibit several biological properties related to neurological and psychiatric conditions (86, 87). Several studies have provided mechanistic insight into the advantageous effects of glucosinolates on brain health through modulating the hypothalamic–pituitary–adrenal axis, reducing neuroinflammation, oxidative stress, beta-amyloid, and tau production, inhibiting DNA methyltransferases, and increasing brain-derived neurotrophic factor and cellular lifespan (88–90). In a clinical study, daily oral administration of isothiocyanate sulforaphane increased peripheral and brain glutathione, an antioxidant, in healthy human subjects (91). In addition, Ghazizadeh-Hashemi et al. showed that a 6-week sulforaphane administration safely decreased depressive symptoms in patients with a history of cardiac interventions and presence of mild to moderate depression (92). Although, a variety of animal model studies has reported a good effectiveness of sulforaphane interventions for psychiatric and neurodegenerative disorders such as depression, anxiety, schizophrenia, cognitive function, learning and memory, multiple sclerosis, and AD (86, 93–96). Therefore, considering that the positive impacts of cruciferous vegetables and their metabolites are mainly associated with antioxidant and anti-inflammatory mechanisms, the ND may also have beneficial outcomes on neurological function.

Apples and pears are widely consumed in the Nordic regions and are generally recognized as healthy food sources (97). Some studies demonstrated that apples are a good source of antioxidants with various phytochemical compounds. The main phytochemicals in apples are flavonoids (including quercetins, catechin, epicatechin, and proanthocyanidins) and phenolic acids (including p-coumaric acid, chlorogenic acid, and caffeic acid) (97). Quercetin, (3,5,7,3′,4′-pentahydroxyflavone), plays a principal neuroprotective role by inhibiting the proinflammatory mediators that are released by glial cells. Indeed, it is a strong scavenger of free radicals such as ROS and reactive nitrogen species (98). It has been proposed that quercetin prevents neuroinflammation by inhibiting the production of NO in glial cells, which further inhibits the NF-κB signaling pathway and prevents inflammation-related neuronal damage (99). Similarly, it ameliorated activated astrocytes which mediate the generation of ROS and cytokines and further influence neuronal cells and degeneration (100). Most importantly, quercetin may protect the neuronal cells against amyloid beta peptide toxicity by activating the nuclear factor (erythroid-derived 2)-like 2 (Nrf2)- antioxidant response elements (ARE) pathway (101). Activation of the Nrf2-ARE signaling pathway promotes neuroprotection against oxidative injury and cell death (102). Another area of interest regarding the neuroprotective mechanisms provided by quercetin is sirtuins. Quercetin has been shown to activate sirtuin-1, which leads to the protection of cells from apoptosis (101).

### 5.2. Whole grains

The ND is characterized by a high content in whole grain cereals versus refined grains. Whole wheat, oat, rye, and barley are among the main food sources of carbohydrates and other nutrients such as dietary fibers, minerals, and vitamins in the Scandinavian nations. It has been reported that whole-grain cereals are high in phytochemicals such as phytosterols, phenolic acid, and β-glucans which are responsible for the high antioxidant properties (103). A Finnish study revealed that whole-grain consumption was very high among the subjects in Finland with the lowest quartile median consumption (79 g/day), which was higher than the highest quintile mean consumption (45.6 g/day) found in the US population (32). There is ample evidence to support that whole grain cereals have health-benefits effects (30). Mounting evidence demonstrated that whole-grain cereals are good sources of fermentable carbohydrates, including resistant starch, dietary fibers, and oligosaccharides, that cannot usually be broken down by the digestive enzymes of humans. However, the gut microflora selectively breaks down and ferments them into gases and short-chain fatty acids (SCFAs) (104). SCFAs are absorbed and utilized by the gastrointestinal epithelial cells, as well as beneficial bacteria such as *lactobacilli* and *bifidobacterial*; therefore improving the gut microbial community, decreasing the gut pH, and enhancing the immunity of the body (105). Extensive research efforts have indicated that an appropriate increase in whole-grain intake (barley and oats) can enhance the growth of probiotics and enhance intestinal and distal organ functions (106, 107). On the other hand, evidence is emerging supporting the role of intestinal microbiota in the brain function by the gut-brain axis, thereby regulating neurological activities such as behavior, cognitive function, and mood (108, 109). Gut-brain axis involves a complex structure of immunological, neural, and endocrinological factors, that can be an important target for manipulating brain health (109). A cross-sectional study demonstrated an inverse relationship between fiber supplementation and depressive symptoms (110). However, there was no significant association in Gopinath et al. study (111). Overall, there are complex mechanistic processes involved in the impacts of dietary fiber-derived whole grains on brain health. Metabolites from bacterial fermentation, such as SCFAs, may play a variety of regulatory roles including histone acetylation, signaling through G protein-coupled receptors, and affect the host’s immune system by altering the circulating levels of inflammatory mediators and reducing neuroinflammation (112). In animal models of endotoxin-induced sickness behavior, pectin as the only source of fiber decreased brain TNF-α and IL-1β (113).

**Figure 3:**
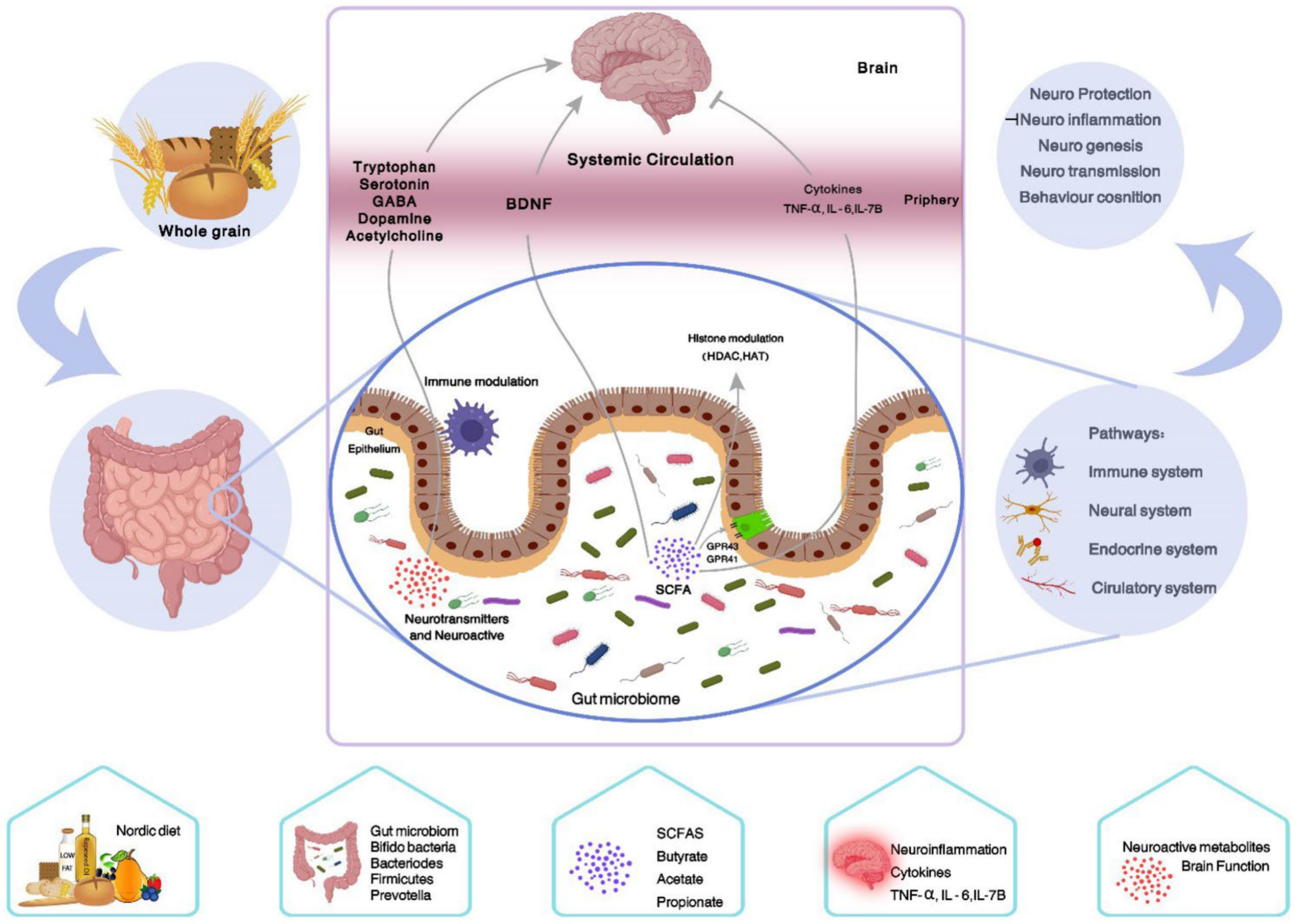
Summary of the effects of the ND on the gut-brain axis. One of the components of the Nordic diet is whole grains, which are a good source of fermentable carbohydrates. The intestinal microbial flora selectively ferments them into gases and SCFAs, which in several ways lead to the improvement of the gut barrier function and effect on the central nervous system (gut-brain-axis). (1) Modulating histone through GPR, (2) reducing cytokines such as TNF-α, IL-6, and IL-1β, (3) modulating neurotransmitters and neuroactive (tryptophan, serotonin, GABA, dopamine, and acetylcholine), (4) increasing BDNF. GABA, Gamma-Aminobutyric Acid; BDNF, Brain-derived neurotrophic factor; TNF-α, Tumor necrosis factor-alpha; IL-6, Interleukin-6; IL-1β, Interleukin-1beta; SCFAs, Short chain fatty acids; GPR, G protein-coupled receptor.

Moreover, gut microbiota modulates serotonin concentration, a neurotransmitter, and the metabolism of tryptophan, which is often targeted by anti-depressant medications in the central nervous system (114). Butyrate, one of the SCFAs, can increase the transcripts of brain-derived neurotrophic factor (BDNF), an important molecule for maintaining the integrity of neurons and cognitive function, in the brain (115). It is worth noting that the gut microbiome may directly or indirectly affect the generation of neurotransmitters, such as dopamine, γ-aminobutyric acid (GABA), and acetylcholine which modulate cognition and mood status (109). The potential action mechanisms of the ND on the gut-brain axis are shown in Figure 3.

Besides fibers, a growing body of evidence has revealed that whole-grain cereals are rich in a variety of phytochemicals, including phytic acid, flavonoids, terpenes, coumarins, and phenolic compounds, which have various physiological activities, including hypotensive, antibacterial, anti-hyperglycemia, immunomodulation, antioxidant, anti-tumor, anti-inflammation, and anti-aging (103). These compounds are documented to exert antioxidant and anti-inflammatory activities, resulting in a neuroprotective effect (103). It is worth noting that studies on animals have shown that diets high in carbohydrates and low in protein could lead to the longest lifespan and improved cognition. It may be concluded that the quality of dietary carbohydrates is more important than its quantity on nerve function and brain health (116, 117).

### 5.3. Legumes

Legumes are nutritionally valuable, providing several nutritious components including proteins with essential amino acids, minerals, vitamins, dietary fibers, complex carbohydrates, unsaturated fats, and non-nutritional compounds such as alkaloids, isoflavone, phytic acid, phenolic compounds (tannins), and saponins (113). Dietary isoflavones derived from legumes may have beneficial effects on brain health, receiving new attention regarding neuroprotective properties (118). These compounds are classified as phytoestrogens which are most extensively investigated. Isoflavones can mimic the transcriptional effects of estrogen *via* binding to estrogen-receptor beta (ERβ), mediating estrogen action, and thereby inhibiting apoptotic cell death (119). Estrogen and its mimics modulate synaptic plasticity, neuronal survival and growth, and brain function through an estrogen receptor-mediated pathway, and subsequently regulation of gene transcription and second messenger systems (120).

Moreover, the antioxidant action of isoflavones has been suggested to be involved in the presumed effect of legumes on brain health. These compounds afford protection against neuroinflammation by ameliorating oxidative damage, upregulating endogenous antioxidant signaling pathways, and protecting the integrity of the mitochondria (119, 121). These antioxidant activities may be attributed to the inhibition of the NF-κB signaling pathway (121). In this regard, some studies demonstrated that isoflavones prevent microglial activation and inflammation (122, 123). *In vitro* studies have shown that isoflavone treatment exhibited protection against apoptosis *via* regulating the antiapoptotic bcl-2 protein suppression and modulating cell survival signaling (120, 121). Other protective effects of dietary isoflavones on brain health can mediate by reductions in proapoptotic proteins such as Bax and Bad, and modulation of kinase signaling (119).

### 5.4. Fish/seafood

Fish, especially fatty fish, and seafood are one of the natural components in the ND, with high amounts of polyunsaturated fatty acids (PUFAs) containing eicosapentaenoic acid (EPA) and docosahexaenoic acid (DHA). In addition, these food sources contain many other valuable nutrients such as vitamins and minerals (30). It was commonly suggested that the major dietary components of fish are DHA and EPA, which are the main building blocks for nerve cell membranes and play a crucial role in proper brain development and function, neurotransmission, and ion channel modulation, and thus are effective in neuroprotection. Some studies reported that there is a relationship between the reduction of omega-3 PUFA concentration in the blood serum and erythrocyte membranes with several neuropsychiatric disorders such as depression, schizophrenia and also dyspraxia, dyslexia, autism, and attention-deficit hyperactivity disorder (ADHD) (124–126). Omega-3 PUFAs have several mechanisms of action in the cerebral vascular system and brain that could improve brain function (127). First, they seem to attenuate inflammation by reducing the production of pro-inflammatory cytokines from arachidonic acid. Omega-3 PUFAs compete with arachidonic acid for incorporation into phospholipid membranes in the cyclooxygenase pathway by preventing the generation of proinflammatory eicosanoids, including prostaglandin E2 and thromboxane B2. EPA and DHA can also prevent the secretion of other pro-inflammatory mediators including interleukins (IL-1, IL-1β, IL-2, IL-6), TNF-α, and interferon-gamma (IFN-γ). Other anti-inflammatory mechanisms are the reduction of T-cell proliferation and inhibition of leukocyte migration. Considering that inflammation is involved in the pathophysiology of neurological disorders, these fatty acids might exert neuroprotective properties through their anti-inflammatory effects (127–129). Second, long-chain omega-3 fatty acids decrease cardiovascular risk factors, including hypertriglyceridemia, and enhance the blood flow in the brain, thus reducing cerebrovascular disease by modulating risk factors (127).

Moreover, a growing body of evidence suggests that omega-3 fatty acids affect the receptor function, the neurotransmitter level, and its metabolism. In this regard, some studies indicated that these fatty acids are related to the activity of the serotoninergic system. There is a relationship between low DHA levels and low concentrations of 5-hydroxyindolacetic acid (5-HIAA), the main metabolite of serotonin, in the cerebrospinal fluid, observed in people suffering from depression and schizophrenia. This can imply that low levels of omega-3 fatty acids are associated with impaired serotonergic neurotransmission (130, 131). DHA induces BDNF synthesis, thus sustaining the life of neurons. In addition, PUFAs deficiency may impair the release and uptake of neurotransmitters, enzymatic processes, and function of ion and receptor channels such as dopaminergic, GABAergic, and cholinergic in nervous system cells, contributing to the development of psychiatric disorders including depression, anxiety, and aggression (126, 132). Some studies suggest that low consumption of fish is associated with increased prevalence of depression (129).

It is also known that the consumption of omega-3 PUFAs is recommended for the prevention of AD. Consuming fish once a week decreases the risk of AD by 60%. EPA and DHA can reduce amyloid-β production and its accumulation in plaques, and improve its clearance (127). In the Cardiovascular Health and Cognition study, consumption of fatty fish (≥ 2 servings per week) reduced the incidence of dementia by 28% and reduced the risk of AD by 41% (133).

### 5.5. Rapeseed oil

Rapeseed oil is regarded to be a key oilseed product in Nordic countries. It is characterized by a low level of saturated fatty acids (7%), and substantial amounts of mono- and polyunsaturated fatty acids such as alpha-linolenic acid, linoleic acid, and oleic acid (134). These fatty acids are known as neuroactive molecules (135). Oleic acid (18:1ω9 monounsaturated fatty acid) is critical for the survival of neural stem cells, which are the source of newborn neurons in the brain and are involved in mood, memory, learning, and stress response. In a rodent model study, oleic acid bound to the orphan nuclear receptor TLX/NR2E1 (nuclear receptor subfamily 2, group E, member 1), converted it from a transcriptional repressor to a transcriptional activator of cell-cycle and neurogenesis genes and induced neurogenesis and brain development (136). Similar beneficial effects were also shown in another animal study, in which oleic acid administration demonstrated its neuroprotective effects in transient and permanent focal cerebral ischemia (137). It is believed that oleic acid is associated with the onset of neurodegeneration disease through anti-inflammatory and vascular activities (138).

Moreover, multiple regression analysis of cohort study data revealed that daily consumption of oleic acid has a significantly positive effect on cognitive decline in community-dwelling Japanese elderly subjects (139). Certain dietary patterns, such as MD which contains high amounts of oleic acid, have been strongly associated with protection against age-related cognitive decline (140–142). Therefore, it is highly likely that rapeseed oil, as a source of oleic acid and PUFAs, may be a beneficial food that is closely related to neurological disorders.

## 6. Strengths and limitations

To the best of our knowledge, this is the first review study demonstrating the potential differential effects of ND on brain function in Nordic versus non-Nordic countries. This is important when considering promoting the adoption of the ND in non-Nordic countries to improve neurological function. Our review also discussed the association of several aspects of the ND with brain health and neurological function that may be less well-regarded. Moreover, what differentiates this review is that it was not limited to a specific neurological function and included research that investigated different neurological domains. This review had some limitations. The methods of dietary assessment and neurological function tests varied from one study to another, which is considered confounding factors.

## 7. Conclusion

Knowing the ingredients and nutritional content of the food we eat, provides important insight into how diet affects our brain health. Several aspects of the ND may lead to some health benefits suggesting neuroprotective effects. High consumption of fruits, vegetables, whole grains, legumes, rapeseed oil, fish, and seafood leads to the high ingestion of dietary phytochemicals, antioxidants, fibers, and mono- and polyunsaturated fatty acids in the ND pattern. The current state of knowledge attributes the possible effects of characteristic components of the ND to its antioxidant, anti-inflammatory, lipid-lowering, gut-brain-axis modulating, and ligand activities in cell signaling pathways. Based on existing evidence, the ND may be considered a recommended dietary approach for the improvement of neurological function and brain health.

### 7.1. Future directions

Future directions should focus on precise dietary patterns and their applications in brain health. The neurological benefits of ND are not very well investigated. On the other hand, considering the different designs of studies and limitations in the number of studies, we chose the narrative approach for the present review. There is a need to investigate the randomized trial in placebo versus Nordic diet group in different populations. Overall, more and long-term human studies are needed to confirm the neuroprotective effects of the ND.

## Data availability statement

The original contributions presented in the study are included in the article/supplementary material, further inquiries can be directed to the corresponding author.

## Author contributions

RJ and VB researched the latest knowledge and publications in the field and wrote the article. VB designed, supervised, and corrected the article. All authors approved the final version of this article for publication.

## Conflict of interest

The authors declare that the research was conducted in the absence of any commercial or financial relationships that could be construed as a potential conflict of interest.

## Publisher’s note

All claims expressed in this article are solely those of the authors and do not necessarily represent those of their affiliated organizations, or those of the publisher, the editors and the reviewers. Any product that may be evaluated in this article, or claim that may be made by its manufacturer, is not guaranteed or endorsed by the publisher.

## Glossary

PD: Parkinson’s disease
AD: Alzheimer’s disease
ND: Nordic diet
MD: Mediterranean diet
NF-κB: Nuclear factor-kappa B
ROS: Reactive oxygen species
TNF-α: Tumor necrosis factor-α
COX-2: Cyclooxygenase-2
PI3K: Phosphoinositide 3-kinase
MAPKs: Mitogen-activated protein kinases
NO: Nitric oxide
PGE2: Prostaglandin E2
IL: Interleukin
SCFAs: Short-chain fatty acids
BDNF: Brain-derived neurotrophic factor
GABA: γ-aminobutyric acid
PUFAs: Polyunsaturated fatty acids
DHA: Docosahexaenoic acid
EPA: Eicosapentaenoic acid
IFN-γ: Interferon-gamma
